# An empyema caused by *Streptococcus constellatus* in an older immunocompetent patient: Case report

**DOI:** 10.1097/MD.0000000000027893

**Published:** 2021-11-12

**Authors:** Young Joo Lee, Jeonghun Lee, Byung Su Kwon, Youngsun Kim

**Affiliations:** a Department of Obstetrics and Gynecology, Kyung Hee University Medical Center, Kyung Hee University College of Medicine, Seoul, Korea; b Department of Internal Medicine, Ye Hospital, Anyang, Korea.

**Keywords:** elderly individuals, empyema, immunocompetence, *Streptococcus constellatus*

## Abstract

**Rationale:**

Empyema caused by *Streptococcus constellatus* is rare in patients without underlying diseases. However, the importance of the *Streptococcus anginosus* group, which consists of *S constellatus*, *S anginosus*, and *Streptococcus intermedius*, as causative organisms of empyema has been increasing.

**Patient concerns:**

A 78-year-old man initially presented with dyspnea and chills for 4 days. He had no medical history.

**Diagnosis:**

Chest X-ray and chest computed tomography showed a large and multiloculated pleural effusion with an air bubble on the right side. Cultivation of the pleural effusion using clone library analysis of the 16S rRNA gene revealed *S constellatus* positivity.

**Interventions:**

The patient was treated by drainage of the pleural effusion and intravenous ceftriaxone and clindamycin for the possibility of anaerobes, followed by 10 weeks of oral antibiotics.

**Outcomes:**

On the 11th day of admission, the thoracic drainage tube was removed. After 1 year of treatment, there were no sequelae of empyema.

**Lessons:**

Although *S constellatus* can cause serious infections in patients with underlying diseases and immunosuppression, physicians need to consider *S constellatus* infection in community-acquired empyema in elderly individuals. It should be treated with early pleural drainage and antibiotics to avoid surgical decortication and prolonged hospitalization.

## 1 Introduction

*Streptococcus constellatus* is a gram-positive, facultative anaerobe, and catalase-negative bacterium, which together with *Streptococcus anginosus* and *Streptococcus intermedius* constitutes the *S anginosus* group (SAG). The SAG, formerly known as the *Streptococcus milleri* group, is a group of commensal bacteria found in the oropharynx, upper respiratory tract, gastrointestinal tract, and urogenital tract mucosa.^[1]^ It is often difficult to evaluate SAG bacteria as causative pathogens. However, they can cause serious infections in patients with immunosuppression or those undergoing invasive procedures.

Empyema is a bacterial infection of the pleural space that results in abscess formation. It is a serious infection with high morbidity and mortality rates. The incidence of empyema has been increasing worldwide, especially during the coronavirus disease 2019 outbreak.^[2,3]^ Treatment often involves invasive procedures such as thoracotomy for pleural decortication, chest tube insertion for drainage, and antibiotic therapy. Early diagnosis of empyema is important for successful treatment. Empyema caused by the SAG mostly occurs in patients with a history of alcohol abuse, immunosuppression, and underlying diseases. Among the SAG bacteria, *S anginosus* is more common, while *S intermedius* is more invasive than *S constellatus*.^[1,4]^ Here, we have reported a case of a 78-year-old immunocompetent man with empyema caused by *S constellatus* who underwent thoracic drainage with antibiotic treatment.

## 2 Case report

A 78-year-old man without underlying diseases was admitted to our hospital with a history of dyspnea and chills for 4 days. He rarely consumed alcohol in recent years and did not smoke. The patient's oral hygiene was good.

**Figure 1:**
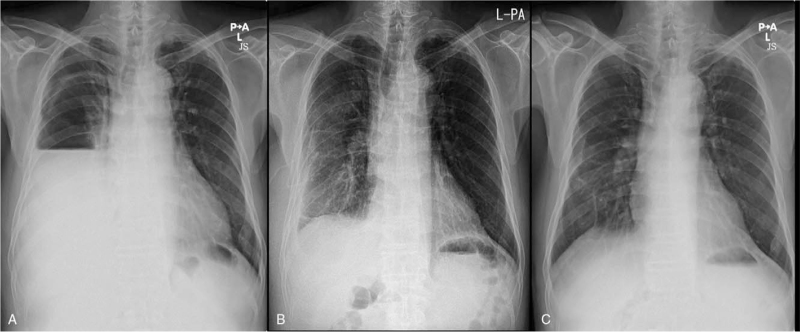
Chest X-ray findings. (A) Massive pleural effusion and a collapsed lung in the right lower lung field on admission. (B) Improved pleural effusion after thoracic drainage on the 11th day of admission. (C) Resolution of the empyema after 1 year.

**Figure 2:**
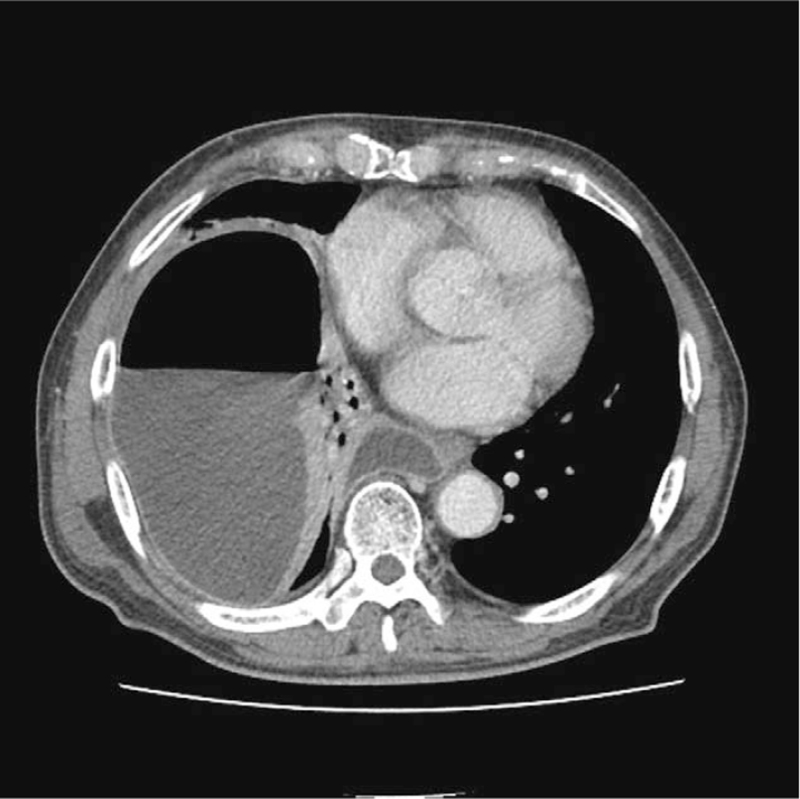
Chest computed tomography findings. Multilobulated pleural effusion and air-fluid level in the right pleural space.

On admission, his blood pressure was 130/80 mm Hg, body temperature was 36.7°C, pulse rate was 72 beats/min, and respiration rate was 22 breaths/min. Lung examination revealed decreased breath sounds and tactile fremitus in the right lung. Complete blood count test results revealed the following: white blood cell (WBC) count, 16,400/μL (segmented neutrophils 88.4%); hemoglobin level, 13.1 g/dL; hematocrit level, 37.6%; platelet count, 394,000/μL; and erythrocyte sedimentation rate, 85 mm/h. Serum biochemical assay results revealed the following:

C-reactive protein, 39.67 mg/dL; protein level, 5.8 g/dL; albumin, 3 g/dL; total bilirubin, 1.11 mg/dL; alkaline phosphatase, 226 mg/dL; aspartate transaminase/alanine transaminase, 78/99 IU/L; blood urea nitrogen, 32.5 mg/dL; creatinine, 0.67 mg/dL; and lactate dehydrogenase, 262 U/L. Chest radiography (Fig. 1A) and chest computed tomography (Fig. 2) showed a large and multiloculated pleural effusion with an air bubble on the right side. Subsequently, the patient underwent right thoracic drainage with thoracentesis. A turbid, yellowish, and macroscopically purulent fluid with an unpleasant odor was drained. ultures were requested. Laboratory analysis of the pleural fluid showed a large number of pyogenic cells, pH 7.0; WBC count, 40,640/μL; red blood cell count, 2100/μL; polymorphonuclear neutrophil count, 92%; lactate dehydrogenase, 4647 IU/L; glucose, 3 mg/dL; protein level, 0.53 g/dL; and adenosine deaminase, 2.2 IU/L

Intravenous empiric piperacillin/tazobactam was commenced. The pleural fluid was drained for 10 days. The blood and sputum culture results showed negative findings. Cultivation of the pleural effusion using clone library analysis of the 16S rRNA gene identified the presence of *S constellatus*, which was sensitive to penicillin, cefotaxime, clindamycin, and vancomycin, but not tetracycline. According to the antibiotic susceptibility results, antibiotic therapy was changed to intravenous ceftriaxone 1 g/day and clindamycin 1800 mg/day during 2 weeks for the coverage of all anaerobes. On the 11th day of admission, 3 L of pleural effusion was drained. The thoracic drainage tube was removed because the drainage volume had reduced and the loculated nature of empyema had decreased (Fig. 1B). The patient's symptoms and laboratory test results (WBC count, 7100/μL C-reactive protein level, 8.90 mg/dL; and erythrocyte sedimentation rate, 48 mm/h) showed improvement. The patient was discharged and prescribed oral antibiotic therapy (cefpodoxime and clindamycin) for 10 weeks. One year after treatment, repeat chest X-ray showed resolution of empyema (Fig. 1C).

This study was approved by the ethics committee and institutional review board of Kyung Hee University Medical Center, Seoul, South Korea. The patient provided informed consent for publication of this case.

## 3 Discussion

The SAG was first described by Guthof in 1956 after being isolated from dental abscesses and other oral inflammatory lesions.^[5]^*S constellatus* belongs to the SAG and is a gram-positive coccus, a facultative anaerobe, and catalase negative. It is a commensal bacterium found in the tissues and organs of various body systems. It is usually not considered a pathogen and may be underestimated, especially in patients without underlying diseases. However, *S constellatus* tends to cause pyogenic infections and has variable clinical manifestations at various sites.^[4]^ Treatment usually includes prolonged antibiotic therapy or surgical interventions.

Culture is the most common diagnostic method for the detection of bacteria. However, the SAG has various biochemical and serological characteristics, and the results are often similar among the SAG species. Therefore, it is difficult to identify the specific bacterium using ordinary aerobic culture media.^[6]^ For this purpose, a 16S rRNA gene sequencing analysis is indicated. In our case, the blood and sputum culture results showed negative findings. We successfully detected *S constellatus* using clone library analysis of the 16S rRNA gene, although only gram-positive bacteria were detected during ordinary cultivation of the pleural effusion.

Empyema is a collection of pus in the pleural cavity. The most common clinical symptoms include dyspnea, fever, chest pain, and cough, which are rarely accompanied by hemoptysis. Laboratory results show an increased neutrophil count and pleural effusion in the form of exudates. The virulence of the SAG is associated with its capsular materials, which contribute to the pathogenicity of the SAG in patients with empyema.^[7]^ Empyema caused by the SAG can infect the lungs through aspiration of oral secretions, direct transmission by trauma or surgery, transmission from surrounding tissues, and blood circulation from other parts of the body.^[8]^ Pneumonia, thoracic surgery, malignancy, diabetes, neurologic disease, alcohol abuse, and mucosal damage (sinusitis and periodontal disease) are known risk factors for empyema.^[9,10]^ Traditionally, the most predominant factor has been assumed to be bacterial pneumonia, but the bacterial etiology of empyema is not necessarily similar to that of pneumonia. The most common causative agents of empyema are *Streptococcus pneumoniae*, *Streptococcus pyogenes*, and *Staphylococcus aureus*. However, the prevalence of SAG as causative organisms has been increasing.^[11]^

Among the SAG species, *S anginosus* is the causative pathogen in most infections, but *S constellatus* is the most commonly found pathogen in chest infections. *S intermedius* is associated with pyogenic infections, while *S anginosus* and *S constellatus* are associated with bacteremia.^[1,4]^ The progression of infection caused by *S constellatus* is generally less severe and is sufficiently treated with pleural drainage and antibiotics. However, *S constellatus* infection may require surgery for decortication in immunocompromised patients.^[12]^*S constellatus* infections should not be underestimated, especially in patients with underlying diseases.

The SAG tends to co-infect with other anaerobes. Co-infection with the SAG and obligate anaerobes is commonly detected in patients with odontogenic infections.^[13]^ This concomitancy leads to increased virulence. Therefore, it is necessary to detect the presence of other anaerobes in empyema caused by the SAG. Patients with empyema often require invasive procedures for pleural decortication, drainage, and antibiotic treatment. In our case, the patient was treated with cephalosporin and clindamycin for 10 weeks. For similar cases, physicians should consider the inclusion of antibiotic treatment.

We have reported a case of an older immunocompetent patient who presented with empyema caused by *S constellatus* and was successfully treated with thoracic drainage and antibiotics. Although empyema caused by *S constellatus* is rare in patients without underlying diseases, it should be considered in community-acquired empyema. For effective treatment, physicians should consider early pleural drainage and antibiotics to avoid surgical decortication and prolonged hospitalization.

## Author contributions

**Conceptualization:** Youngsun Kim, Young Joo Lee, Jeonghun Lee, Byung Su Kwon.

**Investigation:** Youngsun Kim.

**Project administration:** Young Joo Lee, Jeonghun Lee, Byung Su Kwon.

**Resources:** Jeonghun Lee.

**Software:** Young Joo Lee, Jeonghun Lee, Byung Su Kwon.

**Supervision:** Youngsun Kim.

**Writing – original draft:** Youngsun Kim, Young Joo Lee, Jeonghun Lee, Byung Su Kwon.

**Writing – review & editing:** Youngsun Kim.
